# Metasecretome analysis of a lignocellulolytic microbial consortium grown on wheat straw, xylan and xylose

**DOI:** 10.1186/s13068-015-0387-8

**Published:** 2015-12-01

**Authors:** Diego Javier Jiménez, Mukil Maruthamuthu, Jan Dirk van Elsas

**Affiliations:** Department of Microbial Ecology, Groningen Institute for Evolutionary Life Sciences, University of Groningen, Nijenborgh 7, 9747AG Groningen, The Netherlands

**Keywords:** Enzyme cocktail, Metasecretome, Microbial consortium, Glycosyl hydrolases, *Sphingobacterium*, Wheat straw, Xylan, Xylose

## Abstract

**Background:**

Synergistic action of different enzymes is required to complete the degradation of plant biomass in order to release sugars which are useful for biorefining. However, the use of single strains is often not efficient, as crucial parts of the required enzymatic machinery can be absent. The use of microbial consortia bred on plant biomass is a way to overcome this hurdle. In these, secreted proteins constitute sources of relevant enzyme cocktails. Extensive analyses of the proteins secreted by effective microbial consortia will contribute to a better understanding of the mechanism of lignocellulose degradation.

**Results:**

Here, we report an analysis of the proteins secreted by a microbial consortium (metasecretome) that was grown on either wheat straw (RWS), xylose or xylan as the carbon sources. Liquid chromatography–tandem mass spectrometry was used to analyze the proteins in the supernatants. Totals of 768 (RWS), 477 (xylose) and 103 (xylan) proteins were identified and taxonomically and functionally classified. In RWS, the proteins were mostly affiliated with *Sphingobacterium*-like consortium members (~50 %). Specific abundant protein clusters were predicted to be involved in polysaccharide transport and/or sensing (TonB-dependent receptors). In addition, proteins predicted to degrade plant biomass, i.e. endo-1,4-beta-xylanases, alpha-l-arabinofuranosidases and alpha-l-fucosidases, were prominent. In the xylose-driven consortium, most secreted proteins were affiliated with those from Enterobacteriales (mostly *Klebsiella* species), whereas in the xylan-driven one, they were related to *Flavobacterium*-like ones. Notably, the metasecretomes of the consortia growing on xylose and xylan contained proteins involved in diverse metabolic functions (e.g. membrane proteins, isomerases, dehydrogenases and oxidoreductases).

**Conclusions:**

An analysis of the metasecretomes of microbial consortia originating from the same source consortium and subsequently bred on three different carbon sources indicated that the major active microorganisms in the three final consortia differed. Importantly, diverse glycosyl hydrolases, predicted to be involved in (hemi)cellulose degradation (e.g. of CAZy families GH3, GH10, GH43, GH51, GH67 and GH95), were identified in the RWS metasecretome. Based on these results, we catalogued the RWS consortium as a true microbial enzyme factory that constitute an excellent source for the production of an efficient enzyme cocktail for the pretreatment of plant biomass.

**Electronic supplementary material:**

The online version of this article (doi:10.1186/s13068-015-0387-8) contains supplementary material, which is available to authorized users.

## Background

Recently, important developments have paved the way for the use of plant biomass (e.g. wheat straw) as a source of sugars, which—in turn—are useful for the production of bioethanol, plastics and pharmaceutical–chemical intermediates [1]. However, the bioconversion of plant biomass to sugars is a prime bottleneck with respect to efficiency. Next to the need for a physicochemical treatment that affects the complex bonds in lignocellulosic matter, the action of effective degradative enzymes is required. In the latter process, synergism between various enzymes (e.g. oxidases, xylanases, arabinofuranosidases, cellobiohydrolases, endoglucanases and beta-glucosidases) seems to be a “conditio-sine-qua-non” for an efficient process [2, 3]. In the light of process parameters, lignocellulose-degradative enzymes are required to work under diverse conditions, including high temperatures, low or high pH and the presence of residual pretreatment chemicals and inhibitors [4]. Given the difficulty of working with high diversities of required enzymes, plant biomass hydrolysis by microbial consortia instead of single strains has been proposed [5–8]. However, a clear disadvantage of this strategy is that the sugars released from the lignocellulose will be immediately consumed by concurring microorganisms. To overcome this hurdle, enzymes that are released by the microbial consortia into the medium may be applied directly on plant biomass or be used as a supplement to commercial cellulolytic enzyme cocktails. Gladden et al. [9] used a secreted protein fraction of a compost-derived microbial consortium to saccharify pretreated switchgrass, demonstrating that this strategy has excellent potential for the development of (hemi)cellulolytic enzyme cocktails. Additionally, Park et al. [10] reported the development of a cellulase cocktail by combining thermophile-secreted endoglucanases produced by a microcrystalline cellulose-degrading microbial consortium with a recombinant cellobiohydrolase and beta-glucosidase.

Metagenomics and metatranscriptomics studies provide extraordinary insight into the metabolic potential and expression of enzymes in lignocellulolytic microbial consortia [11, 12]. Moreover, total microbial community proteomics (metaproteomics) and—more specifically—metasecretomics (analysis of the total surface-bound and secreted proteins that make up the “secretome” of a microbial community) have the potential to provide a high-resolution representation of proteins and their dynamics outside of the microbial cells. This allows the identification of proteins that are involved directly in the deconstruction of plant biomass [13, 14]. Recently, several studies have reported the analysis of secreted proteins in cultures of single microbial strains. For example, *Ganoderma lucidum* cultivated in sugarcane bagasse [15], *Thermobifida fusca* bred on different lignocellulosic biomass (e.g. corn stover and wood chips) [16], *Aspergillus fumigatus* grown in the presence of glucose, avicel and rice straw [17] and *Ceriporiopsis subvermispora* during growth on aspen wood [18]. However, little is known about the proteins secreted from a wheat straw-degrading microbial consortium across carbon sources with distinct complexities. The presence and abundance of proteins in complex secretomes would guide us in our search for key enzymes that are useful for biorefining. In addition, assessment of the taxonomic affiliation of the secreted proteins enables us to identify the metabolically active microbes, helping to correlate specific functions with the taxa that are truly involved in plant biomass degradation.

Here, we report an analysis of the secreted proteins (metasecretome) from a phylogenetically stable soil-derived microbial consortium [19] that was grown in mineral medium with 1 % of either wheat straw (RWS), xylan or xylose as the unique carbon sources. The selection of untreated instead of pretreated wheat straw was based on the contention that this substrate better selects microbes with high capacity to degrade complex and recalcitrantly bound (hemi)cellulose structures. In addition, the selection of xylan and xylose, as comparators, was based on the composition of the wheat straw. Thus, xylan is a major component of the hemicellulose in wheat straw, xylose being the monomer and unique component of xylan. The metasecretomes were harvested after 11 days of incubation at pH 7.2 under mesophilic aerobic conditions. The analyses indicated that *Sphingobacterium*-like organisms are major players in the degradation of wheat straw, as they were found to secrete key enzymes such as endo-1,4-beta-xylanases (GH10), alpha-l-arabinofuranosidases (GH51) and alpha-l-fucosidases (GH95). Moreover, pullulanases from *Klebsiella*-like organisms were detected in the RWS consortial metasecretome. In contrast, all secreted proteins in the xylose-driven consortial metasecretome were tracked to Enterobacteriales, suggesting that the presence of a pentose like xylose increases the prevalence of bacteria belonging to this class. This study, identified the key enzymes that are predicted to be particularly involved in the attack on wheat straw in comparison to xylan and xylose, giving clues as to the design of enzyme cocktails for future applications.

## Results

### Microbially enriched cultures and metasecretome extraction

**Fig. 1:**
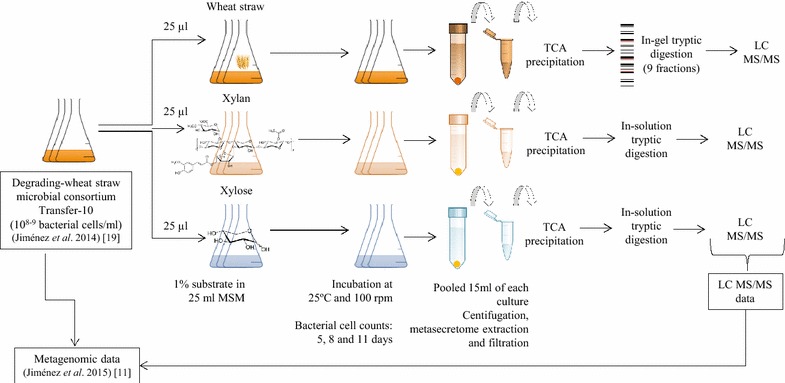
Schematic representation of the methodology used in this study

Microbially enriched cultures were produced with cells from an RWS-derived source consortium [8, 19]. Flasks containing 25 ml mineral medium and 1 % of wheat straw (RWS), xylan or xylose were inoculated with 25 µl of cell suspension (~5 log bacterial cells/ml in the first enrichment flasks). Bacterial growth was found to differ along the 11 days of incubation (Fig. 1). In the RWS-driven consortium, the densities increased from the inoculum level to around 8.5 log bacterial cells/ml after 5 days and then to 9 log bacterial cells/ml after 11 days. However, for the xylan- and xylose-driven consortia, we observed a maximum peak of growth after 5 days (~7.4 and 7.8 log bacterial cells/ml for xylan and xylose, respectively) and relatively stable communities afterwards (~7.0 and 7.6 log bacterial cells/ml for xylan and xylose, respectively, after 11 days of incubation) (Additional file 1).

The secreted proteins (metasecretome) of each treatment were recovered from 15 ml pooled final cultures. Selection of the sampling point (11 days) was based on the assumption that some of the highly recalcitrant compounds in the wheat straw might have been sufficiently degraded, allowing enzyme systems to work on the lignocellulose moieties of the substrate. Proteins in 2 ml of supernatant were precipitated by the trichloroacetic acid (TCA) method. Prior SDS-PAGE analysis of these fractions showed a high complexity of protein bands in the RWS compared with the xylose- and xylan-driven consortia. Given this result, we performed an in-gel tryptic digestion of nine fractions for the RWS treatment, selecting proteins of sizes between 20 and 150 kDa (Additional file 1). Moreover, the secreted proteins from the xylose and xylan treatments were digested with trypsin (in solution) and subsequently used for liquid chromatography–tandem mass spectrometry (LC–MS/MS) (Fig. 1).

### Metasecretomes: overview of the data

**Fig. 2:**
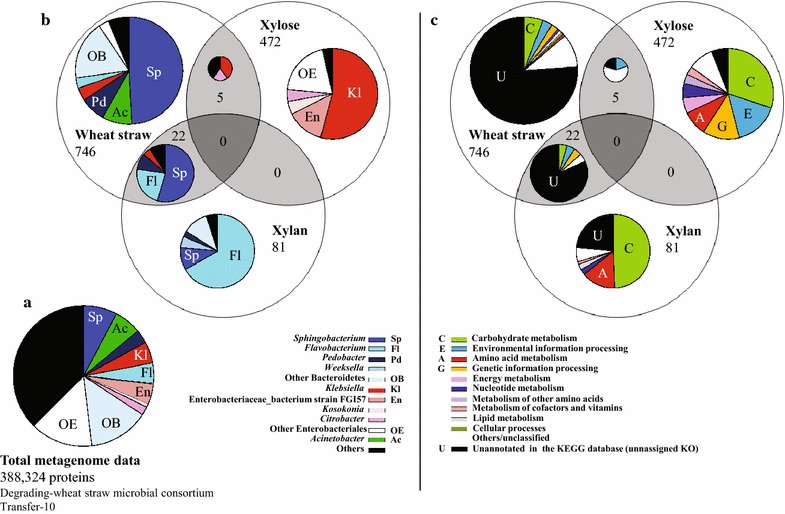
Taxonomic and functional assignment of proteins. **a** Taxonomic assignment of detected proteins in the metagenome database and **b** in each metasecretome, **c** functional assignment of proteins detected in each metasecretome

Totals of 3652, 2402 and 459 peptide sequences, from RWS, xylose and xylan, respectively, were detected and identified from the filtered LC–MS/MS data. Based on database searches (affiliation of the detected peptide sequences to the 388,324 predicted metagenome proteins from the original wheat straw-degrading microbial consortium) [11], 768 (RWS), 477 (xylose) and 103 (xylan) proteins were identified as traceable to the metagenome data. Of these total proteins, only 22 were common between RWS and xylan and five between RWS and xylose. We did not find any common proteins between the xylan and xylose consortial metasecretomes and also not between all three systems (Fig. 2).

### Taxonomic and functional affiliation of secreted proteins

The secreted proteins, in each system, and the total proteins from the database (metagenome) were analyzed using the KEGG database on the GhostKOALA platform (GHOSTX threshold: 100) [20] (Fig. 2). Overall, 8, 6, 5, 5 and 3 % of the predicted proteins from the metagenome were affiliated to proteins from *Sphingobacterium*, *Acinetobacter*, *Flavobacterium*, *Klebsiella* and *Pedobacter*-like organisms, respectively (Fig. 2a). In the RWS consortial metasecretome, most of the proteins were affiliated with proteins detected in Bacteroidetes, specifically 48 % *Sphingobacterium*, 8 % *Pedobacter*, 3 % *Flavobacterium* and 18 % “others”. In addition, 8 and 4 % of the proteins were affiliated with those from *Acinetobacter* and *Klebsiella*-like organisms, respectively. In the metasecretome from the xylose-grown consortium, approximately 96 % of the proteins were assigned to predicted proteins encoded by the genomes of different Enterobacteriales (i.e. 54 % *Klebsiella*, 13 % Enterobacteriaceae bacterium strain FGI57, 5 % *Kosakonia*, 4 % *Citrobacter* and 20 % “others”). Finally, the predominance (~95 %) of Bacteroidetes-related proteins was found in the metasecretome of the xylan-driven consortium, among which 56 % were similar to proteins produced by *Flavobacterium*-like organisms. Thus, a shift in the secreted proteome was found, depending on the carbon source (Fig. 2b).

Regarding the functional annotation, around 86 % of all proteins detected in the RWS metasecretome were unannotated/unclassified based on the KEGG orthology system, thus representing as-yet-undescribed proteins with potential function (Fig. 2c). However, the remaining proteins in this metasecretome were mostly classified within the KEGG functional classes (orthology groups) carbohydrate metabolism (6 %), environmental information (3 %) and genetic information (2 %) processing. In contrast, in the metasecretome of the xylose-driven consortium, 86 % of the proteins were classifiable based on the KEGG orthology system, the most abundant functions being related with carbohydrate metabolism (30 %), environmental information (16 %) and genetic information processing (13 %). In the metasecretome of the xylan-grown consortium, 49 and 15 % of the proteins were classified within the classes carbohydrate and amino acid metabolism, leaving 29 % of unannotated/unclassified proteins plus 7 % low-abundance classes (Fig. 2c).

### Analysis of proteins detected in the metasecretomes: abundant functions

The unique proteins detected in the RWS (746), xylose (472) and xylan (81) metasecretomes were—separately—compared to each other, and clustered, at 70 % of amino acid identity. This allowed to infer the “abundant” clusters (clusters containing >2–3 detected proteins) of proteins for each metasecretome. Thus, we could discern 38 (RWS), 38 (xylose) and 11 (xylan) abundant protein clusters (more than 3 protein sequences per cluster in RWS and xylose; and more than 2 proteins per clusters in xylan) (Table 1; Additional files 2, 3). In addition, 408, 243 and 53 singletons (i.e. clusters containing only a single protein sequence) were found in the RWS-, xylose- and xylan-driven metasecretomes, respectively.

**Table 1:** Abundant clusters (clusters at 70 % identity with more than 3 sequences) of secreted proteins in RWS

Cluster	# PS	LRS	Best PSI-BLAST hit vs. NCBI (organism source)	Accession No.	COV (%)	*e* value	Identity	Domain ID	Domain description
1	7	161	Hemagglutinin (*Sphingobacterium*)	WP_045753147.1	76	8E−74	90	pfam01832	Mannosyl-glycoprotein endo-beta-*N*-acetylglucosaminidase
2	6	159	RNA methyltransferase (*Sphingobacterium*)	KKX48125.1	100	3E−110	100	pfam00588	SpoU rRNA methylase family
3	6	136	Flagellin (*Enterobacter*)	WP_042717930.1	100	4E−84	98	pfam00669	Bacterial flagellin
4	6	155	Membrane protein (*Sphingobacterium*)	WP_045755907.1	100	5E−94	90	pfam14322	Starch-binding associating with outer membrane (SusD)
5	6	161	Pyruvate dehydrogenase (*Sphingobacterium*)	WP_021189278.1	100	6E−111	100	pfam02780	Transketolase
6	6	83	Membrane protein (*Sphingobacterium*)	WP_045753095.1	100	1E−40	94	TIGR04056	TonB-linked outer membrane protein, SusC/RagA family
7	6	153	Outer membrane lipoprotein (*Acinetobacter*)	WP_004695452.1	96	5E−104	100	pfam03548	Outer membrane lipoprotein carrier protein LolA
8	6	154	Molecular chaperone GroEL (*Sphingobacterium*)	WP_045754355.1	100	2E−94	99	PRK00013	Chaperonin GroEL
9	5	149	Hypothetical protein (*Sphingobacterium*)	WP_021190406.1	100	6E−90	94	TIGR04056	TonB-linked outer membrane protein, SusC/RagA family
10	4	153	Aminopeptidase (*Sphingobacterium*)	WP_045755761.1	100	3E−107	99	cd02619	C1 peptidase family, also referred to as the papain family
11	4	155	Hypothetical protein (*Sphingobacterium*)	WP_038697635.1	98	9E−95	86	pfam06283	Trehalose utilization
12	4	131	Hypothetical protein (*Sphingobacterium*)	WP_021189714.1	100	1E−74	85	cd02968	Synthesis of cytochrome c oxidase
13	4	154	Hypothetical protein (*Sphingobacterium*)	WP_021188014.1	55	6E−49	98	pfam11138	Protein of unknown function (DUF2911)
14	4	153	Hypothetical protein (*Sphingobacterium*)	WP_021192491.1	88	8E−88	98	pfam13472	GDSL-like lipase/acylhydrolase family
15	3	85	Hypothetical protein (*Sphingobacterium*)	WP_037526146.1	97	1E−48	99	ND	ND
16	3	135	Alpha-l-arabinofuranosidase (*Sphingobacterium*)	WP_045756399.1	100	1E−88	99	pfam06964	Alpha-l-arabinofuranosidase
17	3	144	Hypothetical protein (*Sphingobacterium*)	KKX50961.1	100	5E−94	100	TIGR04056	TonB-linked outer membrane protein, SusC/RagA family
18	3	153	Hypothetical protein (*Sphingobacterium*)	WP_045755931.1	62	1E−52	86	ND	ND
19	3	162	Pyruvate dehydrogenase (*Sphingobacterium*)	WP_021192396.1	100	6E−102	99	TIGR01349	Pyruvate dehydrogenase
20	3	82	TonB-linked outer membrane protein (*Sphingobacterium*)	WP_003012895.1	84	1E−21	70	TIGR04056	TonB-linked outer membrane protein, SusC/RagA family
21	3	137	Membrane protein (*Klebsiella*)	WP_032728567.1	61	1E−48	98	PRK10554	Outer membrane porin protein C
22	3	122	Flagellin (*Alicycliphilus*)	WP_013723065.1	100	3E−68	95	pfam00669	Bacterial flagellin
23	3	152	Porin (*Serratia*)	WP_024529021.1	100	5E−99	96	pfam02264	LamB porin, maltoporin
24	3	157	Hypothetical protein (*Sphingobacterium*)	KKX47093.1	87	2E−83	99	TIGR04056	TonB-linked outer membrane protein, SusC/RagA family
25	3	162	Superoxide dismutase (*Flavobacterium*)	WP_041515972.1	93	2E−95	92	pfam00080	Copper/zinc superoxide dismutase
26	3	140	Hypothetical protein (*Sphingobacterium*)	WP_045754573.1	100	2E−84	99	COG1729	Periplasmic TolA-binding protein (function unknown)
27	3	90	Flagellar motor protein MotB (*Sphingobacterium*)	WP_038696344.1	82	2E−36	86	ND	ND
28	3	97	Pyruvate dehydrogenase (*Sphingobacterium*)	WP_045753967.1	100	6E−60	100	pfam00198	2-oxoacid dehydrogenases acyltransferase
29	3	71	Pullulanase (*Klebsiella*)	EHS90708.1	100	4E−41	96	TIGR02103	Alpha-1,6-glucosidases, pullulanase-type
30	3	162	Hypothetical protein (*Sphingobacterium*)	WP_021191620.1	100	4E−110	99	pfam07980	SusD family
31	3	82	Hypothetical protein (*Acinetobacter*)	WP_005399834.1	100	1E−50	99	pfam03724	META domain; unknown function, some are secreted
32	3	83	Hypothetical protein (*Acinetobacter*)	WP_004981111.1	86	2E−35	86	ND	ND
33	3	145	Hypothetical protein (*Sphingobacterium*)	KKX46588.1	100	4E−85	93	TIGR04056	TonB-linked outer membrane protein, SusC/RagA family
34	3	151	Pullulanase (*Klebsiella*)	EHS90708.1	100	2E−100	95	TIGR02103	Alpha-1,6-glucosidases, pullulanase-type
35	3	156	PhoH-like ATP-binding protein (*Salmonella*)	WP_001731743.1	100	3E−106	100	COG1702	Phosphate starvation-inducible protein PhoH, predicted ATPase
36	3	83	Gliding motility protein RemB (*Sphingobacterium*)	WP_038696674.1	97	4E−37	81	ND	ND
37	3	151	Aldose 1-epimerase (*Sphingobacterium*)	KKX49924.1	100	6E−97	95	cd09019	Galactose mutarotase_like
38	3	154	Membrane protein (*Sphingobacterium*)	WP_038697956.1	100	1E−95	95	TIGR04056	TonB-linked outer membrane protein, SusC/RagA family

*#PS* number of protein sequences, *LSR* length of representative sequence in amino acids, *COV* query coverage, *ND* not identified

On the basis of the PSI-BLAST annotation, the representative sequences of the abundant clusters, in the RWS-driven metasecretome, were mostly classified as hypothetical proteins (14 clusters), membrane proteins (5), proteins involved in motility (e.g. flagellin) (4), pyruvate dehydrogenases (3) and proteins involved in polysaccharide deconstruction (e.g. pullulanases and alpha-l-arabinofuranosidases) (3). Moreover, RNA methyltransferases and chaperones like GroEL were found (Table 1). Domain detection using the NCBI conserved domain database revealed that the hypothetical and membrane proteins (19 of 38 abundant clusters: 50 %) might reflect plant polysaccharide sensors/transporters such as TonB-dependent receptors. Interestingly, the most abundant protein cluster was affiliated with a hemagglutinin from *Sphingobacterium*-like organisms. Such proteins contain a pfam01832 domain, which is related with a mannosyl-glycoprotein endo-beta-*N*-acetylglucosaminidase (EC:3.2.1.96) (Table 1).

In the xylose-driven metasecretome, we observed high abundances and diversities of proteins involved in several different pathways (Additional file 2). The high-abundance protein clusters (38) were related to functions mostly dealing with sugar metabolism (e.g. fructose-bisphosphate aldolases, xylose isomerases and xylulose kinases) (5 clusters), next to ribosomal proteins (3). In addition, some oxidoreductases, dehydrogenases and ABC transporters were detected. Regarding the xylan-driven metasecretome, the most abundant protein clusters encompassed proteins such as glycine dehydrogenases (cluster 1 with 5 sequences), hydroperoxidases (cluster 2 with 4 sequences) and xylose isomerases (cluster 3 with 3 sequences) from *Flavobacterium*-like organisms (97 % amino acid identity) (Additional file 3).

A total of 22 proteins was common between the RWS and xylan systems. These were mostly affiliated with Bacteroidetes-like membrane-localized proteins (mainly involved in transport, polysaccharide-binding and motility). In addition, four aldehyde dehydrogenases, two hypothetical proteins and one superoxide dismutase were detected. Moreover, one pullulanase from *Klebsiella*-like organisms (97 % identity) was common in the RWS- and xylan-driven consortium metasecretomes (Additional file 4).

### Overview of carbohydrate-active proteins detected in the three metasecretomes

**Fig. 3:**
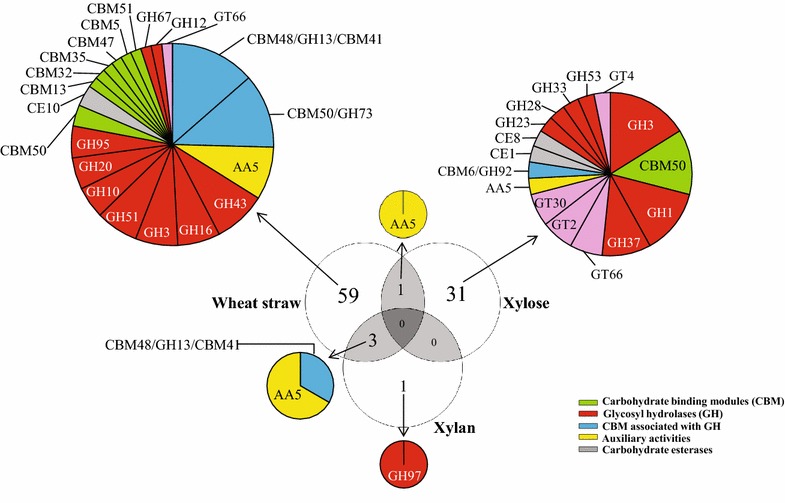
Carbohydrate-active annotation of proteins detected in each metasecretome

All proteins detected in the three metasecretomes were also annotated based on the CAZy database [21]. Fifty-nine, thirty-one and one unique proteins from the RWS-, xylose- and xylan-driven metasecretomes, respectively, were homologous (*e* value threshold: 0.01) to particular carbohydrate-active enzymes (Fig. 3). Based on this annotation, in the RWS consortial metasecretome, proteins of the CAZy families CBM48/GH13/CBM41 (14 %) and CBM50/GH73 (12 %) were the most abundant (proteins with 2 or 3 CAZy modules), followed by AA5 (glyoxal/galactose oxidases) and glycosyl hydrolases (GHs) of CAZy families GH43, GH16, GH3, GH51, GH10, GH20, GH95, GH12 and GH67. In the xylose metasecretome, the proteins were mostly affiliated with those from the CAZy families GH1 (16 %), CBM50 (13 %), GH3 (13 %) and GH37 (10 %). In addition, we observed the presence of glycosyl transferases (GTs) of families GT66, GT2, GT30 and GT4. The one protein identified in the xylan-driven metasecretome was classified as a presumptive GH97 family protein. This protein was affiliated, based on PSI-BLAST, with an alpha-glucosidase from *Flavobacterium* sp. F52 (WP_008466439—100 % coverage and 98 % identity).

### Plant biomass-degrading proteins detected in the wheat straw metasecretome

The proteins annotated as carbohydrate-active enzymes in the RWS metasecretome were manually analyzed and re-annotated based on the PSI-BLAST hits (Table 2). Using this analysis, we observed eight proteins (RWS1–8) containing CBM48/GH13/CBM41 modules. These were all affiliated with two pullulanases from *Klebsiella*-like organisms (identity from 95 to 100 %). In addition, the proteins detected within CAZy families GH43 (5), GH51 (4), GH16 (4), GH3 (4), GH10 (3), GH95 (3), GH12 (1) and GH67 (1) (Fig. 3) were identified as GH43A/D19 precursor (probably a beta-xylosidases) (RWS21–25), alpha-l-arabinofuranosidases (RWS26–29), glycerophosphodiester phosphodiesterases (RWS13–16), putative beta-glucosidases (RWS17–20), endo-1,4-beta-xylanases (RWS9–11), alpha-l-fucosidases (RWS31–33), peptidases (RWS12) and alpha-glucuronidases (RWS30), respectively. All these last 25 proteins were affiliated with *Sphingobacterium*-like sequences, showing high identity and coverage (>70 %). In addition, proteins affiliated with those of CAZy family AA5 were detected, of which two were tracked to the Gammaproteobacteria, specifically *Pseudomonas* and *Acinetobacter* species. The PSI-BLAST analyse had catalogued these as “membrane/flagellar proteins” (RWS34–38) (Table 2).

**Table 2:** Plant biomass-degrading enzymes found in the RWS metasecretome

Protein number	CAZy family	Action on (activity)^a^	Best PSI-BLAST hit vs. NCBI (organism source)	Accession no.	COV (%)	*e* value	Identity (%)	COV2	#*P*
RWS1	CBM48|GH13|CBM41	Alpha-glucan polysaccharides (alpha-amylase/pullulanase)	Pullulanase (*Klebsiella*)	EHS90708.1	100	3E−45	98	43	5
RWS2	CBM48|GH13|CBM41		Pullulanase (*Klebsiella*)	WP_017144955.1	100	8E−48	100	37	3
RWS3	CBM48|GH13|CBM41		Pullulanase (*Klebsiella*)	EHS90708.1	100	9E−34	97	33	3
RWS4	CBM48|GH13|CBM41		Pullulanase (*Klebsiella*)	EHS90708.1	100	4E−41	96	28	3
RWS5	CBM48|GH13|CBM41		Pullulanase (*Klebsiella*)	EHS90708.1	100	2E−64	99	45	3
RWS6	CBM48|GH13|CBM41		Pullulanase (*Klebsiella*)	EHS90707.1	100	1E−91	98	34	4
RWS7	CBM48|GH13|CBM41		Pullulanase (*Klebsiella*)	WP_017144955.1	100	2E−41	97	48	5
RWS8	CBM48|GH13|CBM41		Pullulanase (*Klebsiella*)	EHS90708.1	100	2E−100	95	30	3
RWS9	GH10	Hemicellulose (endoxylanase)	XynB19 precursor (*Sphingobacterium*)	ACX30647.1	100	9E−47	95	57	5
RWS10	GH10		Endo-1,4-beta-xylanase precursor (*Sphingobacterium*)	ACX30652.1	100	4E−67	95	38	3
RWS11	GH10		Endo-1,4-beta-xylanase precursor (*Sphingobacterium*)	ACX30652.1	98	1E−101	92	27	5
RWS12	GH12	Cellulose (endoglucanase)	Peptidase M16 (*Sphingobacterium*)	WP_045752104.1	100	3E−96	97	17	2
RWS13	GH16	(Hemi)cellulose (endoglucanase/endogalactanase)	Glycerophosphodiester phosphodiesterase (*Sphingobacterium*)	WP_031286917.1	100	2E−70	99	55	5
RWS14	GH16		Glycerophosphoryl diester phosphodiesterase (*Sphingobacterium*)	KKX48537.1	100	7E−50	100	34	3
RWS15	GH16		Glycerophosphoryl diester phosphodiesterase (*Sphingobacterium*)	KKX48537.1	100	5E−77	100	24	3
RWS16	GH16		Glycerophosphodiester phosphodiesterase (*Sphingobacterium*)	WP_031286917.1	100	2E−95	99	41	5
RWS17	GH3	Cellulose (beta-glucosidase)	Beta-glucosidase (*Sphingobacterium*)	CDS95530.1	100	7E−106	95	21	3
RWS18	GH3		Glycoside hydrolase family 3 (*Sphingobacterium*)	KKX49060.1	81	1E−52	92	27	2
RWS19	GH3		Glycoside hydrolase family 3 (*Sphingobacterium*)	KKX49060.1	100	9E−94	92	21	3
RWS20	GH3		Glycoside hydrolase family 3 (*Sphingobacterium*)	KKX49060.1	75	3E−34	96	36	2
RWS21	GH43	Hemicellulose (beta-xylosidase/alpha-arabinofuranosidase)	Aldose epimerase (*Sphingobacterium*)	WP_045756400.1	100	4E−81	81	11	2
RWS22	GH43		GH43D19 precursor (*Sphingobacterium*)	ACX30655.1	100	3E−83	98	29	3
RWS23	GH43		GH43D19 precursor (*Sphingobacterium*)	ACX30655.1	100	1E−51	100	37	2
RWS24	GH43		GH43D19 precursor (*Sphingobacterium*)	ACX30655.1	97	8E−40	94	38	2
RWS25	GH43		GH43A19 precursor (*Sphingobacterium*)	ACX30649.1	100	3E−88	98	15	2
RWS26	GH51	Hemicellulose (alpha-arabinofuranosidase)	Alpha-l-arabinofuranosidase (*Sphingobacterium*)	WP_045756399.1	100	1E−88	99	27	2
RWS27	GH51		Alpha-l-arabinofuranosidase (*Sphingobacterium*)	WP_045756399.1	92	5E−45	99	44	2
RWS28	GH51		Alpha-l-arabinofuranosidase (*Sphingobacterium*)	WP_038697950.1	100	3E−69	96	33	2
RWS29	GH51		Alpha-l-arabinofuranosidase (*Sphingobacterium*)	WP_045756399.1	100	3E−52	100	43	2
RWS30	GH67	Hemicellulose (alpha-glucuronidase)	Alpha-glucuronidase (*Sphingobacterium*)	WP_031288024.1	90	5E−69	83	15	2
RWS31	GH95	Hemicellulose (alpha-fucosidase)	Alpha-l-fucosidase (*Sphingobacterium*)	WP_045753034.1	99	8E−72	76	37	4
RWS32	GH95		Alpha-l-fucosidase (*Sphingobacterium*)	WP_045753034.1	100	1E−44	82	46	3
RWS33	GH95		Alpha-l-fucosidase (*Sphingobacterium*)	WP_045753034.1	100	5E−71	77	37	4
RWS34	AA5	Lignin or polysaccharides (glyoxal/galactose oxidases)^b^	Cell envelope biogenesis protein OmpA (*Flavobacterium*)	WP_042565523.1	100	2E−44	99	18	2
RWS35	AA5		Outer membrane protein omp38 (*Acinetobacter*)	WP_004819145.1	66	7E−53	98	39	5
RWS36	AA5		Flagellar motor protein MotB (*Sphingobacterium*)	WP_031288562.1	100	4E−82	98	24	4
RWS37	AA5		Flagellar motor protein MotB (*Sphingobacterium*)	WP_038696344.1	98	4E−53	97	65	6
RWS38	AA5		Membrane protein (*Pseudomonas*)	WP_016485176.1	100	8E−81	100	29	3

*COV* query coverage, *COV2* coverage based on the peptides sizes in each protein, *#P* number of peptides per protein

^a^Activities predicted by CAZy database [21, 37]

^b^Copper radical oxidases able to oxidize carbohydrates, aldehydes, alcohols with generation of H_2_O_2_ [37]

## Discussion

Complete degradation of plant biomass depends on the synergistic action of different enzymes. Thus, analysis of the proteins that are secreted by lignocellulose-grown microbial consortia can shed light on the complexity of the proteome involved in degradation. Here, we provide an analysis of the metasecretome of microbial consortia grown on raw wheat straw (RWS), xylan and xylose as the sole carbon sources (Fig. 1). The source microbial consortium bred on wheat straw had already revealed beta-galactosidase, beta-xylosidase, beta-mannosidase, cellobiohydrolase and beta-glucosidase extracellular activities [19]. We observed low consortial growth (~10^7^ bacterial cells/ml), next to a low abundance of secreted/detected proteins (103) on xylan, as compared to RWS (~10^9^ cells/ml and 768 proteins) (Additional file 1). Thus, somewhat unexpectedly, the xylan was less palatable than the wheat straw to the consortium used. Wheat straw likely contains diverse high-complexity as well as low-complexity carbon sources, which collectively provide “niches” that spur the growth of the different microbes that had been “primed” for its use given their preselection on this substrate.

Detection of the proteins in the metasecretomes of each treatment was performed with the help of a metagenomic database obtained from the original microbial consortium [11]. This methodology was found to improve the detection of proteins compared with the use of public databases such as NCBI or UniProt [22]. However, a potential caveat in this process should be mentioned: the proteins detected were mostly fragmentary and so only yielded hits with gene fragments. Also, signal peptides were not detected with the proteins, as reported [16]. To quantify the relative abundance of the proteins, the high-throughput isobaric tag (iTRAQ) technique has been proposed [13, 17]. Alternatively, label-free methods and the “exponentially modified protein abundance index” (emPAI) have been applied [16]. In our study, we applied a label-free method, refraining from using the emPAI. The rationale was that this index may suffer from biases, specifically in the spectrum counting, due to the fact that small proteins tend to have fewer peptides identified per protein compared with large proteins [23]. To fill this gap, we performed a “semi-quantitative” analysis of the secreted proteins using an innovative clustering method, in which the detected proteins (in each treatment) were grouped at 70 % of amino acid identity. Then, we considered clusters having more than three sequences to constitute “abundant” types of proteins. Moreover, in our study, the functional annotation of the proteins was supported by the search for conservative domains in the most abundant protein clusters. This methodology can assist in the assignment of hypothetical proteins to a known function, used by us, for example, in the case of the TonB-dependent receptors (Table 1).

Although the RWS-bred microbial consortium contained bacteria as well as fungi, the abundance of bacteria (estimated as ~10^9^ 16S rRNA gene copies/ml) was higher than the fungi (~10^7^ ITS1 copies/ml) [8]. The majority (around 70 %) of the predicted protein-encoding genes, in the RWS metagenome data, had homologs on published bacterial genomes, which supported the contention of primary selection of bacterially dominated substrate-degrading microbial consortia. In contrast, metagenomic sequences affiliated with the Eukarya amounted to less than 0.001 % of the totals [11]. Based on these premises and due to the fact that the proteins in the metasecretomes of each treatment were identified with the help of a metagenomic database from the original microbial consortium (RWS), it is logical to assume that fungal proteins were present in low abundance and could not be easily detected.

Based on the taxonomic assignment of abundant proteins (Fig. 2), we inferred that different organisms in our consortia have different roles. Specifically, *Sphingobacterium*, *Pedobacter* and *Flavobacterium* types were hypothesized to act mainly on the complex substrates (wheat straw and xylan), whereas *Klebsiella*-like organisms were more tuned to utilizing sugars such as xylose. In contrast, in our original consortium, the latter organisms were surmised to degrade (hemi)cellulose through the expression of endoglucanases, xylanases and glucosidases [8, 11, 19]. Moreover, the detection of TonB-dependent receptors in the RWS metasecretome indicated that sensing of plant polysaccharides and transport of sugars might have been dominant in particular Bacteroidetes-like organisms [24–26]. Genes for such proteins have previously been found to be enriched in the metagenome from the source consortium when compared with that from the microbial source (forest soil) [11]. The most abundant protein cluster, in RWS, showed a domain that belongs to mannosyl-glycoprotein endo-beta-*N*-acetylglucosaminidase from *Sphingobacterium*-like consortium members (Table 1). Such proteins, called autolysins, may be involved in the degradation of peptidoglycan. They are involved in daughter cell separation during vegetative cell growth, hydrolyzing the septum after cell division [27, 28]. Thus, these proteins might not have a primary role in lignocellulose degradation.

Regarding the carbohydrate-active enzymes detected in the RWS metasecretome (Fig. 3; Table 2), *Sphingobacterium*-like organisms might be assumed as being the sources of most of the secreted enzymes involved in cellulose (GH3, GH12) and hemicellulose deconstruction (e.g. GH10, GH43, GH51 and GH95). Recently, Jiménez et al. [11] reported an overrepresentation of genes for family GH3, GH43 and GH95 proteins in the metagenome of the source consortium when compared with that of the forest soil inoculum. Together, these data suggest a correlation between the enrichment of genes for these families, their expression and the concomitant protein secretion. We postulate that this type of selection strongly impacts the wheat straw deconstruction rate. The absence of cellobiohydrolases (CBHs) and endoglucanases (specifically of families GH5 and GH9) in the RWS metasecretome could be related to the consortium response to the composition of the substrate or to their low abundance. Other studies have shown similar results. For instance, D’haeseleer et al. [14] reported the absence of CBHs in the metasecretome of a thermophilic bacterial consortium adapted to switchgrass (JP-1 % SG). They reported the presence of only one abundant and overrepresented protein involved in cellulose hydrolysis (a GH5). In contrast, the majority of secreted GHs were related with the deconstruction of hemicellulose or alpha-glucan polysaccharides (GH13 and GH31). Interestingly, comparison of this metasecretome [14] with that of the same consortium that had been perturbed by cultivation with microcrystalline cellulose (McCel) [10] demonstrated that the GHs complement was different for the McCel-degrading microbial consortium. In particular, one CBH and three endoglucanases were detected in McCel that were not detected in the JP-1 % SG. These data, plus our results, indicate that the lignocellulolytic microbial consortia, and their metasecretomes, respond differently as related to the composition of the substrates used. Additionally, we hypothesized that the microbes in RWS could get energy and act efficiently at the expense of mainly the hemicellulose fractions instead of the microcrystalline cellulose.

Moreover, finding proteins affiliated with CAZy family AA5 (oxidative enzyme—glyoxal/galactose oxidase) may pinpoint potential roles for *Sphingobacterium*, *Acinetobacter* or *Pseudomonas* in this activity. Glyoxal oxidases are copper-radical oxidases with a broad specificity that are able to oxidize aldehydes to the corresponding carboxylic acids. Such proteins are considered to be one of the central H_2_O_2_-generating enzymes. Also, galactose oxidases (GAO) are copper-containing enzymes that catalyze a reaction comprising two separable half-reactions, i.e. oxidation of a primary alcohol, and reduction of O_2_ to H_2_O_2_. GAO catalyze the oxidation of a wide range of carbohydrates (including galactose) but also primary alcohols to the corresponding aldehydes, with the reduction of O_2_ into H_2_O_2_ [37]. Based on that, we hypothesize that such proteins are involved in the oxidation of polysaccharides or in lignin transformations in the RWS metasecretome, possibly improving the deconstruction of the plant biomass.

The finding of eight proteins with CBM48/GH13/CBM41 (pullulanases—EC 3.2.1.41) modules affiliated with those from *Klebsiella*-like organisms (Table 2) pinpointed *Klebsiella* as the source organism for this specific activity. Enzymes of family GH13 (called debranching enzymes) constitute the major glycoside hydrolase family acting on substrates containing alpha-glucoside linkages (alpha-glucan polysaccharides). Pullulanases are a specific class of GH13 glucanases, with type I pullulanases specifically attacking alpha-1,6 linkages and type II pullulanases also alpha-1,4 linkages. Alpha-glucan polysaccharides (e.g. starch, rhamnogalacturonan and (1–6)-alpha-glucomannan) are present in plant cell walls and hence the aforementioned protein may be denoted as plant polysaccharide-degrading enzymes. Pullulanases break down pullulan, an exopolysaccharide produced from starch and other biopolymers [29]. These enzymes are useful in hydrolyzing side chain residues of hemicellulose and other alpha cross-links that could be present in the wheat straw used for breeding [30]. Recently, Adav et al. [16] reported the upregulation of GH13 proteins (alpha-glucosidases or pullulanases) in the secretome of *Thermobifida fusca* when grown on corn stover, hay and wood chips, suggesting that such proteins might enhance the hydrolysis of plant biomass.

Interestingly, D’haeseleer et al. [14] reported the presence of proteins affiliated with CAZy families GH1, GH2, GH3, GH10 and GH51 in the metasecretome of a thermophilic bacterial consortium growing on switchgrass (JP-1 % SG). In addition, endoglucanases (GH5 and GH9), cellobiohydrolases (GH48), xylanases (GH10) and arabinofuranosidases were found in the metasecretome of microcrystalline cellulose-degrading consortia (McCel) [10]. These last studies reflect the importance of proteins from these families to deconstruct plant biomass. Especially hemicellulolytic enzymes such as endo-1,4-beta-xylanases (GH10), beta-xylosidases (GH43), alpha-l-arabinofuranosidases (GH43 or GH51) and alpha-l-fucosidases (GH95) could be indispensable in the formulation of efficient enzyme cocktails. These could be added to cellulose- and alpha-glucan polysaccharides-degrading enzymes, i.e. endoglucanases (GH5), cellobiohydrolases, beta-glucosidases (GH1or GH3) and pullulanases (GH13). The commercial development of hemicellulases for the enzymatic hydrolysis of plant biomass is not as advanced as that of cellulases because current commercial preparations have been primarily used on pretreated biomass from which the hemicellulose part was partially removed before saccharification [4]. Moreover, current fungus-derived cellulases tend to have only weak hemicellulolytic activity and are not adequate for the complete conversion of hemicellulose from plant biomass. Development of low-cost commercial hemicellulases that work synergistically with cellulases is one of the goals of many current research activities [31]. Based on this premise, the hemicellulases secreted by the here-described RWS consortium could constitute an excellent source for the preparation of enzyme cocktails that can be applied directly or as a supplement in plant biomass pretreatment. Complete genes (with start and final codons) for these secreted hemicellulases can be identified in the assembled metagenome files [11] and then might be synthesized, codon-optimized, cloned and expressed, in order to produce the individual enzymes and combine them in cocktails. Recently, four genes from the GH43 family (*Sphingobacterium* origin) were already expressed successfully in *E. coli* from our metagenome data (unpublished observations).

Regarding the xylose consortium metasecretome, the profile of carbohydrate-active enzymes was quite different from that of the RWS one. Clearly, the presence of the xylan monomer xylose repressed the production and release of hemicellulases (e.g. GH43, GH51 and GH95) and pullulanases (GH13). However, oligosaccharide-degrading enzymes (GH1 and GH3) and GTs were detected. Unfortunately, we could not find enzymes directly involved in polysaccharide degradation in the xylan-driven metasecretome, possibly because these were not abundant. However, we did find an alpha-glucosidase (GH97) apparently from a *Flavobacterium*-like organism as well as enzymes related with the conversion (isomerization) of sugars that are release after degradation of xylan (e.g. xylose isomerases). Such sugar isomerases were present in the metasecretomes of both the xylan- and xylose-driven consortia. In previous work, extracellular xylose isomerases have been found in the supernatant of a thermophilic bacterial consortium adapted to switchgrass [14]. These enzymes may have an extracellular role, transforming monosaccharides produced from hemicellulose, potentially as a means of relieving product inhibition on the primary hemicellulases or as a way to circumvent competition for these monosaccharides from other members of the microbial community.

## Conclusions

Analyses of the metasecretomes of lignocellulolytic microbial consortia grown on 1 % of either xylose, xylan or wheat straw revealed several key proteins to become abundant in relation to the substrate used. First, the microbial consortium performed poorly on xylan as compared to xylose and wheat straw. In terms of protein “richness”, the metasecretome of the RWS-driven consortium stood out as the “richest”. In the RWS (as well as xylan) consortium, proteins released by Bacteroidetes (e.g. *Sphingobacterium*, *Flavobacterium* and *Pedobacter*) were dominant. In contrast, proteins secreted by Enterobacteriales abounded in the xylose-driven metasecretome. In this respect, we also found *Klebsiella*-like organisms to secrete pullulanases in the RWS-driven consortium.

The highly diverse RWS metasecretome revealed an abundance of CAZy-defined GH3, GH10, GH13, GH43, GH51, GH67 and GH95 family enzymes, which are all predicted to be involved in plant biomass breakdown. The fact that these proteins were mainly affiliated with *Sphingobacterium*-like enzymes suggested sphingobacteria are highly relevant players in the degradation of lignocellulose. Thus, an intricate relationship between the active consortial members was shown, providing evidence of synergistic activities of the different proteins in the deconstruction of plant biomass. We advocate the use of the RWS consortium released proteins as potential enhancers of efficiencies in plant biomass treatments. Future experiments that combine metasecretomes, other hemicellulases (e.g. synthesized from the metagenome) with available commercial cellulases can help to improve the plant biomass degradation rates for biorefining. Finally, an analysis of the secreted proteins during consortium development (time course) posits as very valuable, allowing to observe the differential expression and secretion of the enzymes involved in plant biomass deconstruction.

## Methods

### Microbial enriched cultures design

The original lignocellulose decomposing consortium was developed with a forest soil as the microbial source inoculum [8]. Briefly, the soil-derived cell suspensions were introduced into triplicate flasks containing 25 ml of mineral salts medium (MSM) with 1 % of “raw” wheat straw (RWS), and incubated at 25 °C with shaking at 100 rpm. A dilution-to-stimulation approach was followed up to the 10th transfer [8, 19]. Then, 25 µl (1 × 10^8^ bacterial cells/ml) were transferred from the 10th transfer flask to three flasks (in triplicate) containing sterile and fresh MSM plus 1 % (w/v) of different carbon sources, i.e. xylose, xylan (from beechwood) and wheat straw. Subsequently, all flasks were incubated at 25 °C with shaking at 100 rpm. The numbers of bacterial cells per milliliter were quantified by microscope cell counting in a *Burke*-*Turk* chamber (Blaubrand^®^) according to the manufacturer’s protocol. After 11 days of incubation, the microbial cells as well as particulate matter were removed by centrifugation (7500*g* for 30 min at 4 °C). The supernatants (from 15 ml of pooled culture in each treatment) were subsequently filtered by 0.45 µm (Whatman FP30/0.45—cellulose acetate membrane) (in order to remove wheat straw particles) followed by 0.22 µm syringe filters (Whatman FP30/0.22—cellulose acetate membrane) (in order to remove microbial cells) (Fig. 1).

### Metasecretome recovery and liquid chromatography–tandem mass spectrometry

A total of 2 ml of filtered supernatant (secreted proteins) for each treatment were precipitated with tri-chloroacetic acid (TCA) (final concentration of 15 %). The samples were incubated on ice for 15 min and then overnight at −20 °C. They were then kept at room temperature and proteins were precipitated by centrifugation at 14,000 rpm for 15 min at 4 °C. Following this, supernatants were gently removed, and the tubes spun briefly to remove remaining liquid. The protein pellets were suspended in 1 ml of 100 % ice-cold acetone by five times vortexing for 30 s, over 1 h at −20 °C. Then, samples were centrifuged at 14,000 rpm for 15 min at 4 °C and the acetone was removed, followed by air-drying the pellets for 10 min. Pellets were resuspended with 2× sample buffer (100 mM DTT, 2 % SDS, 80 mM Tris–HCl, pH 6.8, 0.006 % bromophenol blue, 15 % glycerol), after which they were checked with 10 % SDS-PAGE at 80 V for 1.5 h. This was followed by coomassie staining for 6 h in shaking platform at room temperature, and de-staining (40 % methanol and 10 % glacial acetic acid) for 2 h at room temperature.

The secreted proteins from the RWS system were divided into 9 fractions (ranging from 20 to 150 KDa) from the SDS-PAGE gel. These gel fragments were washed with 25 mM of ammonium bicarbonate and 50 % acetonitrile. The xylose and xylan derived samples were also prepared for in-solution digestion. Proteolytic treatment of all samples was performed using trypsin (Promega Benelux BV, Leiden), dissolved in 100 mM ammonium bicarbonate buffer. In the case of the RWS fractions, the gel pieces were mixed with 20 µl of 10 ng/µl trypsin in 100 mM ammonium bicarbonate buffer and incubated overnight at 37 °C. Peptides were extracted from the gel with 75 % of acetonitrile (in 5 % formic acid), dried and resuspended in 20 μl of 0.1 % of formic acid.

After trypsin digestion, the peptide mix was injected into an Ultimate 3000 nano-LC–MS/MS system (Dionex, Amsterdam, The Netherlands), in line connected to an Q-Exactive-Plus-mass spectrometer (Thermo Fisher Scientific, Bremen, Germany). The sample mixture was loaded on a trapping column (Acclaim PepMap; C18; 5-mm length by 300-µm inside diameter; 5-µm particle size; 100-Å porosity; Dionex) and washed. After 3 min, the mixture was separated using a 60-min linear gradient from 5 % of 0.1 % of formic acid in water to 90 % of 0.1 % in acetonitrile at a flow rate of 250 nl/min. The mass spectrometer was operated in data-dependent mode, automatically switching between MS and MS/MS acquisition for the eight most abundant multiple charged ions (2, 3, 4, 5 and 6 times). Full-scan MS spectra were acquired from *m/z* 400 to 1800 in the Q-Exactive-Plus-mass spectrometer at a target value of 3E6 with a resolution of 70,000. Peptides were analyzed in the orbitrap with a resolution of 35,000. The scan range for MS/MS was set to *m/z* 200–2000.

### Peptide identification and data processing

The mass spectrometry datasets produced for each system were analyzed for peptide and protein identification using PEAKS software v7.0 [32]. Spectra for the RWS metasecretomes were pooled (9 spectra). Database searches were carried out against a total metagenome dataset (MG-RAST ID 4547280.3: 388,324 proteins detected by FragGeneScan software) [11]. The following search parameters were applied: (i) trypsin was chosen as the protein-digesting enzyme, (ii) false discovery rate (FDR), 0.1 %, (iii) fragment mass error tolerance 0.02 Da, (iv) peptides with >10 ppm mass error were discarded, (v) proteins with at least two unique peptide matchings were retained, and two missed cleavages were tolerated, (vi) fixed modifications (carbamidomethylation, 57), variable modifications (oxidation—methionine—16). The total metagenome dataset and the detected proteins in each system were taxonomically and functionally classified by the GhostKOALA annotation tool [33] using a cutoff GHOSTX score of 100. Based on this threshold, we predicted the medium–high confidence in the taxonomic affiliation [20]. In order to obtain “semi-quantitative” data, we clustered (threshold of 70 % amino acid identity) the detected proteins using the CD-hit software [34]. Clusters composed of more than three protein sequences (“abundant” proteins) were retrieved. A representative and randomly picked protein sequence for each cluster was manually annotated by PSI-BLAST [35] and inspected for conserved domains using the NCBI conserved domain database. Additionally, the proteins detected in each metasecretome were affiliated with carbohydrate-active enzymes using the CAZymes analysis toolkit (CAT) platform [36] (*e* value threshold 0.01), and obtained hits were manually inspected by PSI-BLAST.

## Additional files

10.1186/s13068-015-0387-8 Bacterial cell counts in the enriched cultures and SDS-PAGE of protein secretomes. **a** Bacterial cell numbers (log10 cells per milliliter of culture) in xylose, xylan and wheat straw after 5, 8 and 11 days of incubation, **b** SDS-PAGE of the secreted proteins by the microbial consortium after 11 days of incubation in xylose, xylan and wheat straw.
10.1186/s13068-015-0387-8 Abundant clusters (clusters at 70 % identity with more than 3 sequences) of secreted proteins in xylose.
10.1186/s13068-015-0387-8 Abundant clusters (clusters at 70 % identity with more than 2 sequences) of secreted proteins in xylan.
10.1186/s13068-015-0387-8 Common proteins between RWS and xylan metasecretomes.

## Abbreviations

AA: auxiliary activities
CAZy: carbohydrate-active enzymes database
CAT: cazymes analysis toolkit
CBM: carbohydrate-binding modules
emPAI: exponentially modified protein abundance index
FDR: false discovery rate
GHs: glycosyl hydrolases
GTs: glycosyl transferases
iTRAQ: isobaric tags for relative and absolute quantitation
KEGG: Kyoto encyclopedia of genes and genomes
LC–MS/MS: liquid chromatography–tandem mass spectrometry
PSI-BLAST: position-specific iterated-BLAST
RWS: wheat straw consortium
SDS-PAGE: sodium dodecyl sulphate-polyacrylamide gel electrophoresis
