# Supplementary figures and images for: Metasecretome analysis of a lignocellulolytic microbial consortium grown on wheat straw, xylan and xylose
Source: Biotechnol Biofuels. 2015 Dec 1;8:199. doi: 10.1186/s13068-015-0387-8 (PMC4666044; doi:10.1186/s13068-015-0387-8)

**A)**

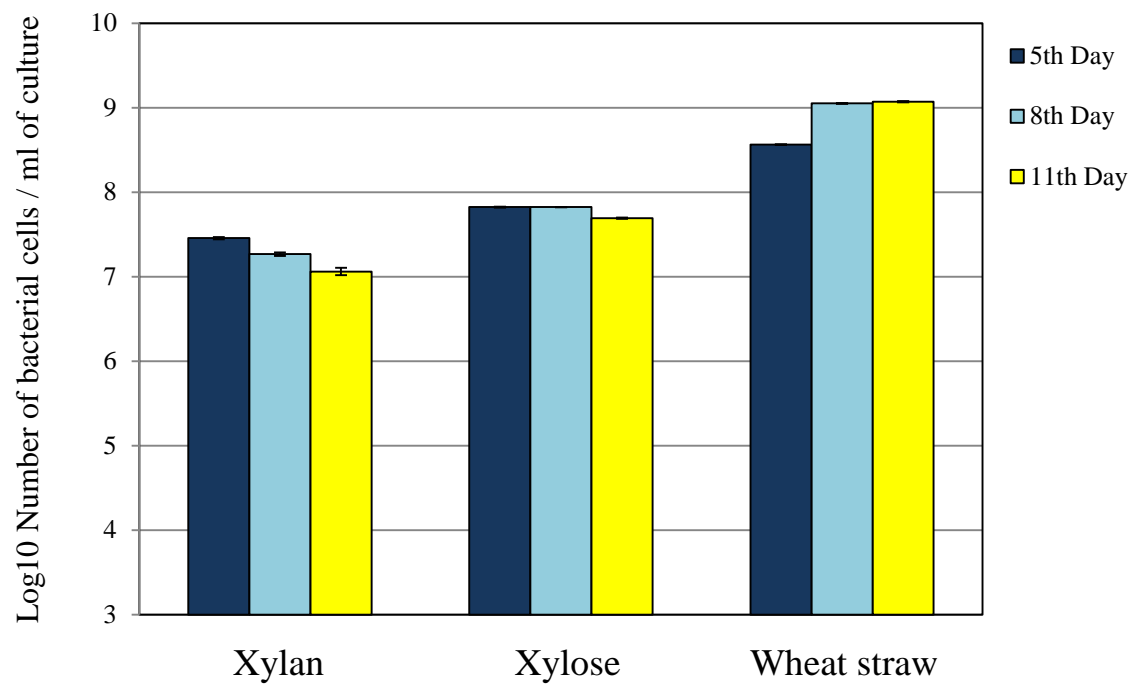

**B)**

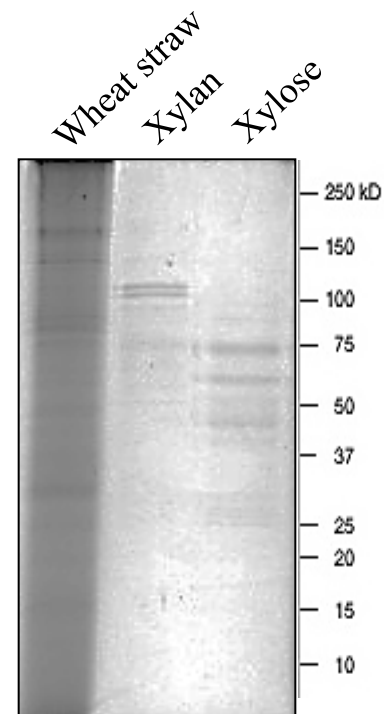

Additional file 1.

Supplement: Supplementary file 1 — 10.1186/s13068-015-0387-8 Bacterial cell counts in the enriched cultures and SDS-PAGE of protein secretomes. a Bacterial cell numbers (log10 cells per milliliter of culture) in xylose, xylan and wheat straw after 5, 8 and 11 days of incubation, b SDS-PAGE of the secreted proteins by the microbial consortium after 11 days of incubation in xylose, xylan and wheat straw. [file 13068_2015_387_MOESM1_ESM.pdf]
